# Evolutionary Studies on the Coxsackievirus A-24 Variants Causing Acute Hemorrhagic Conjunctivitis with Emphasis on the Recent Outbreak of 2023 in India

**DOI:** 10.3390/v17030371

**Published:** 2025-03-05

**Authors:** Sanjaykumar Tikute, Jahnabee Boro, Vikas Sharma, Anita Shete, Alfia Fathima Ashraf, Ranjana Mariyam Raju, Sarah Cherian, Mallika Lavania

**Affiliations:** 1 Enteric Viruses Group, ICMR-National Institute of Virology, 20-A, Dr. Ambedkar Road, Pune 411001, Maharashtra, India; sanjaytikute@yahoo.co.in (S.T.); borojahnabee17@gmail.com (J.B.); 2 Bioinformatics Group, ICMR-National Institute of Virology, 20-A, Dr. Ambedkar Road, Pune 411001, Maharashtra, India; sharmvikas3529@gmail.com (V.S.); alfiyafathimaasharaf@gmail.com (A.F.A.); rmariyamr42@gmail.com (R.M.R.); 3 Maximum Containment Laboratory, ICMR-National Institute of Virology, Sus Road, Pashan, Pune 411001, Maharashtra, India; anitaaaich2008@gmail.com

**Keywords:** AHC, Coxsackievirus A-24v, virus isolation, whole-genome sequencing, phylodynamic, phylogeography, viral evolution

## Abstract

Acute Hemorrhagic Conjunctivitis (AHC) is primarily caused by viral infections, with Coxsackievirus A-24v (CV-A24v) being a significant culprit. Enteroviruses, including CV-A24v, are responsible for global AHC outbreaks. Over time, CV-A24v has evolved, and genotype IV (GIV) has become the dominant strain. This study focused on examining the genetic features and evolutionary trends of CV-A24v responsible for the recent AHC outbreak of 2023 in India. Researchers isolated viral strains from ocular swabs and confirmed the presence of CV-A24v using reverse transcriptase quantitative PCR (RT-qPCR) and whole-genome sequencing. Genomic comparisons between isolates of 2023 and those from a previous outbreak in 2009 were conducted. Phylogenetic analysis revealed that the 2023 isolates formed a distinct cluster within GIV-5 and were related to recent strains from China and Pakistan. The older Indian isolates from 2009 grouped with GIV-3. New subclades, GIV-6 and GIV-7, were also identified in this study, indicating the diversification of CV-A24. Molecular clock and phylogeographic analysis traced the virus’s circulation back to the 1960s, with the common ancestor likely to have originated in Singapore in 1968. The 2023 Indian strains probably originated from Thailand around 2014, with subsequent spread to China and Pakistan. This study concluded that the 2023 outbreak was caused by a genetically distinct CV-A24v strain with nine mutations, underlining the virus’s ongoing evolution and adaptations and offering valuable insights for future outbreak control.

## 1. Introduction

Conjunctivitis is a contagious predominant ophthalmic disease observed globally, affecting individuals of all age groups and socioeconomic backgrounds [1,2]. Viral agents are found to be the predominant cause of the disease, causing approximately 80 percent of the acute cases [3]. Among these infectious agents, viral and bacterial conjunctivitis are the most prevalent, whereas allergen and toxin-induced variants represent non-infectious agents [2]. The disease can be represented by various forms such as Acute Hemorrhagic Conjunctivitis (AHC), epidemic keratoconjunctivitis (EKC), pharyngoconjunctival fever (PCF), and herpetic conjunctivitis, presenting a significant risk of transmission due to its high contagiousness. AHC is predominantly attributed to enteroviruses, enterovirus-D70 (EV-D70) and CV-A24, although adenoviruses are also documented to be associated with the disease [4].

Conjunctivitis outbreaks due to CV-A24v occur every 2–3 years as episodes; since it is highly contagious, the infection spreads rapidly, and most of the population is affected. Therefore, it is required to determine the etiology of the outbreak. Since several episodes of such outbreaks occur, the evolutionary pattern of the viral agent will not be known until compared with the previous CV-A24v viral strains. The clinical manifestations of conjunctivitis involve a variety of ocular discomfort and visual disorders. Patients usually experience a pink/red discoloration in the white of the eye(s), swelling of the conjunctiva and/or eyelids, watery eyes/heavy tearing, and sensation of a foreign object in the eye(s), as well as itching, irritation, and burning sensations or sometimes blurry vision [5,6]. AHC is characterized by a rapid onset of symptoms, quick dissemination and a short incubation period typically ranging from 12 to 18 h [7].

The etiological agents responsible for viral conjunctivitis, such as adenoviruses, rubella viruses, rubeola viruses, herpesviruses, and picornaviruses like human enterovirus-C (HEC) and human enterovirus-D (HED), which include CV-A24 and EV-D70, respectively, contribute to outbreaks with global implications. Enteroviruses belong to *Picornaviridae* family and have a nonenveloped, single-stranded positive RNA genome with an icosahedral capsid, 7500 nucleotides, and 27 nm diameter. Enteroviruses consist of four structural and seven nonstructural proteins, 2A, 2B, 2C, 3A, 3B, 3C, and 3D. There are four structural proteins, VP1 to VP4, of which VP1 to VP3 are located on the surface of the viral capsid and VP4 is located inside the capsid. Human Enterovirus (HEV) is further divided into four subtypes, HEV-A to HEV-D, and CV-A24v belongs to the HEV-C subtype. Specifically, the emergence and spread of CV-A24v leading to AHC outbreaks have been extensively documented, highlighting its widespread geographic distribution and temporal evolution from the initial report in Ghana in 1969 to recent outbreaks in Northern India in 2010 [8]. CV-A24v has the same genome organization as other enteroviruses (EVs). Recently, India along with neighboring countries like Vietnam and Pakistan and other countries like Ghana experienced conjunctivitis outbreaks where the etiology was found to be a variant of CV-A24 belonging to the *Picornaviridae* family [9].

The need for this study arises from the limited molecular characterization and whole-genome data available for recently isolated viral strains from India, particularly during the recent 2023 CV-A24v conjunctivitis outbreak. Understanding the evolutionary patterns of CV-A24v is crucial for identifying circulating genotypes and newly emerging recombinant strains, which could provide insights into the virus’s transmission and mutation dynamics. By comparing the whole-genome sequences of these newly isolated strains with previously reported reference strains, this study aims to shed light on the phylogenetic relationships and genetic evolution of CV-A24v, both in the context of the current outbreak and in comparison to the strain from an earlier conjunctivitis outbreak in 2009. This will help to enhance surveillance efforts and inform strategies for controlling future outbreaks.

## 2. Materials and Methods

### 2.1. Sample Collection and Identification of EV

**Figure 1:**
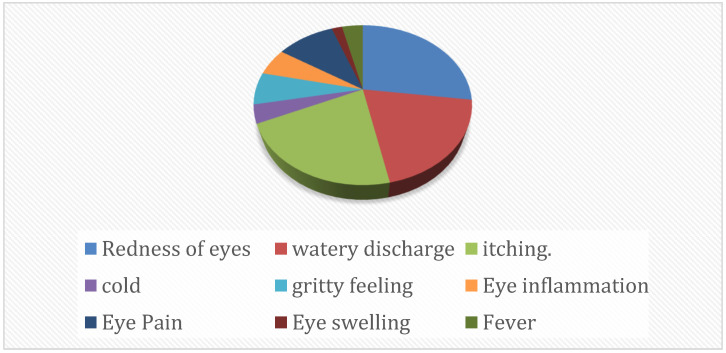
Symptomatic profile of the patients whose samples were selected for virus isolation.

Clinical specimens such as ocular swabs (n = 301) were collected from the outbreak site and also provided by other hospitals for the confirmation of viral etiology along with the informed consent of the patients. The project was approved by the Institutional Ethical Committee (MP-24A-20N). The symptomatic profile revealed eye inflammation with pain, watery discharge, redness, itchiness, and in some cases, blurred vision. Initially, the clinical specimens were screened for the presence of EV by amplification of 5′NCR. The ABI TaqMan 4X Fast Virus Kit (Applied Biosystems, Waltham, MA, USA) was used in a 25 µL reaction with 7 µL of extracted RNA, following recommended cycling conditions. Positive specimens were further analyzed by amplifying the 3C protease gene region for enterovirus typing using primers 3C-F and 3C-R. The 25 µL PCR mixture contained RNA, 2X PCR buffer, SSIII enzyme, primers, and nuclease-free water. The cDNA preparation included incubation at 45 °C for 10 min and 50 °C for 20 min. For PCR amplification, samples were denatured to 94 °C for 2 min, followed by 35 cycles, and the product was purified using QIAquick PCR Purification Kit (Qiagen, Hilden, Germany) and sequenced on an Applied Biosystems sequencer. The positive specimens were further subjected to enterovirus typing based on the 3C protease gene region (3C-F: 5′AAAGGGATGGATCGTCAAGC-3′ and 3C-R: 5′-TAGCCTCTTCAAAGTCTGTC-3′). The samples that showed a threshold Ct value below 25 were specifically selected for virus isolation. The symptomatic profile of the patients whose samples (ocular swabs) were selected to attempt virus isolation is depicted in Figure 1.

### 2.2. Virus Isolation

Samples identified as CV-A24v through molecular characterization were selected for virus isolation. The isolation was undertaken using a susceptible HeLa cell line [10]. A 24-well plate of HeLa cells was prepared with minimum essential medium (MEM Hi-Media, India), supplemented with 10% fetal bovine serum, 100 U/mL penicillin, and 100 µg/mL streptomycin, and incubated at 37 °C in a 5% CO_2_ environment. The clinical specimens were inoculated onto the HeLa cell line, in both undiluted (neat) and 1:10 diluted forms, along with the cell controls [10]. Infected cells exhibiting cytopathic effects (CPEs) were identified and harvested simultaneously, and blind passages were conducted up to passage five (P-5). The P-5 cultures were incubated for 5 days before they were declared negative. 

### 2.3. Confirmation of Isolates by Real-Time q-Reverse Transcriptase PCR (qRT-PCR)

RNA extraction was carried out using a Magmax RNA extraction kit from all the P-5 viral lysates. The RNA was then subjected to EV detection through RT-qPCR with primers targeting the 5′NCR using CFX96TM Real-Time System (Bio-RAD) [11,12]. Pan-Enterovirus quantitative real-time PCR was performed on a CFX-96 real-time apparatus (BioRad, Hercules, CA, USA) to detect enterovirus, amplifying the CV A-24 3C protease region with primers, 3C-F (5′AAAGGGATGGATCGTCAAGC-3′) and 3C-R (5′-TAGCCTCTTCAAAGTCTGTC-3′). The 25 µL PCR reaction mixture contained 5 µL RNA, 12.5 µL 2× PCR buffer, 1.0 µL SSIII enzyme (ThermoFisher Scientific, Waltham, MA, USA), 1.0 µL of each primer (0.2 µM 3C-F/3C-R), and nuclease-free distilled water to reach a final volume of 25 µL. The reaction was incubated at 45 °C for 10 min and 50 °C for 20 min to prepare cDNA, followed by 35 cycles of 30 s at 94 °C, 30 s at 45 °C, and 60 s at 68 °C, with a final extension step at 68 °C for 10 min. The PCR product was purified using the QIAquick PCR Purification Kit (QIAGEN, Hilden, Germany) and sequenced with primers 3C-F and 3C-R using an automated sequencer (Applied Biosystems, Foster City, CA, USA). The sequence was compared to a database of enterovirus serotypes using the BLAST tool (GenBank) to identify the enterovirus type, with a minimum 75% nucleotide similarity threshold required to assign the same serotype.

### 2.4. Whole-Genome Sequencing Using Next-Generation Sequencing (NGS)

RNA was extracted from eight viral strains, including two viral isolates from an earlier 2009 AHC outbreak, using the Magmax RNA extraction kit (ThermoFisher Scientific, Waltham, MA, USA). After measuring RNA concentration, host ribosomal RNA was depleted using the NEBNext rRNA depletion kit (New England Biolabs, Ipswich, MA, USA). The RNA was purified and quantified with Qubit from Thermo Fischer Scientific, USA, while a library was prepared using the TruSeq Stranded mRNA kit (llumina, San Diego, CA, USA), and the integrity of the libraries were examined with the help of Tapestation. The libraries were sequenced on the Illumina Miniseq platform (MiniSeq High Output Kit), and the resulting FASTQ files were analyzed using CLC Genomics Workbench software Version 20 (CLC, Qiagen, Aarhus, Denmark) [12].

### 2.5. Genome Assembly and Annotation

De novo assembly for sequences of eight CV-A24v isolates was performed using Trinity assembler (Version 2.15.2) with default parameters [13]. Subsequently, the assembled genomic sequences were annotated with the help of a viral genome annotation pipeline, VAPiD (v1.6.7)2 [14]. Finally, the eight annotated sequences were deposited to GenBank using BANKIT. 

### 2.6. Sequence Data Collection

A significant amount of CV-A24v sequence data from around the world is available in public databases, and analyzing all these sequences can be time-consuming and also challenging to represent effectively. Complete CV-A24v genomes and VP1 and VP2 nucleotide sequences spanning epidemic years from 1970 to 2024 that were publicly available were downloaded from the GenBank NCBI database in August 2024. Prior to phylogenetic analysis, redundant or highly similar sequences were filtered by clustering sequences with 99.5% similarity or greater, based on location and sampling year using a custom in-house script developed in Visual Basic. The process ensured that representative sequences from each geographic region and outbreak year were selected, minimizing bias from the oversampling of closely related viral strains. The set of sequences for the VP1 gene [15] was utilized, including the relevant closely related sequences from Indian viral strains, to ensure consistency with previous inferences.

### 2.7. Sequences, Phylogenetic and Phylodynamic Analysis

An all vs. all pairwise similarity comparison between the whole-genome sequences was computed using BLAST 2.12.0+ [16]. Multiple sequence alignment was conducted using MAFFT v7.526 [17]. The sequence alignment quality was improved in an automated manner using TrimAl v1.5.rev0 and manual curation [18]. Identification of the best-fit substitution model and maximum likelihood phylogenetic tree was performed using IQ-TREE v2.0.7 [19]. The phylogenetic tree was visualized using ggtree v3.10.1 [20]. Treecluster, a tree-based clustering method, was used for the automatic identification of subgroups within genotype IV (GIV), with a clustering threshold of “0.05” [21]. Phylogeography and molecular clock analysis were conducted using BEASTv1.10.4 with parameters set to be a chain length of 100 million and Bayesian skyline tree priors. The log marginal likelihood values obtained from both path sampling (PS) and stepping stone sampling (SS) were used to decide the suitability of the clock model. A relaxed log normal clock model was implemented as it showed better log marginal likelihood values (-8473.1052 for PS and -8474.3919 for SS) than the strict clock model (-8483.7140 for PS and -8484.5075 for SS). The rate of nucleotide substitution and the 95% highest posterior density (HPD) limits were estimated using tracer v1.10.4 “https://tree.bio.ed.ac.uk/software/tracer/ (accessed on 14 October 2024)”. Transmission routes and migration links were identified using SPREAD v0.9.7.1 software [22]. The maximum clade credibility (MCC) tree was obtained using TreeAnnotator with 10% “burn-in” “https://tree.bio.ed.ac.uk/software/tracer/ (accessed on 14 October 2024)” and visualized using Figtree v1.4.4 (https://tree.bio.ed.ac.uk/software/figtree/, accessed on 14 October 2024).

### 2.8. Mutation Analysis Based on Whole-Genome Sequencing

Comprehensive mutation analysis at the amino acid level was conducted for all available whole-genome sequences from 1970 to 2024, utilizing the 1970 Singapore strain as the reference [23]. The nucleotide sequences were aligned with the prototype strain using MAFFT v7.526 software to identify the nucleotide substitutions [17]. The aligned FASTA file was subsequently analyzed to identify the mutations by employing an in-house Python pipeline. To confirm the derived mutations, we utilized MEGA11, where the aligned FASTA file was translated to assess specific changes at the amino acid level. We examined sequences obtained from ICMR-National Institute of Virology, Pune, India (ICMR-NIV) from the years 2009 and 2023 to uncover novel mutations, if any.

**Figure 2:**
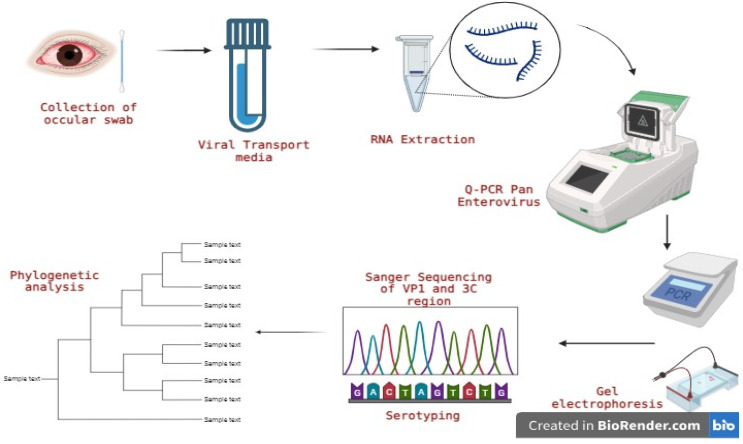
Schematic representation of the workflow.

A schematic representation of the complete workflow from sample processing to analyses is shown in Figure 2.

## 3. Results

### 3.1. Molecular Characterization of Enterovirus-Positive Samples

Among the n = 301 ocular swabs, 158 were found to be EV-positive. All the EV-positive samples were further typed, and 73 were specifically identified as the CV-A24v serotype through amplification and sequencing of the partial 3C protease region. 

### 3.2. Isolation of CV-A24v 

**Figure 3:**
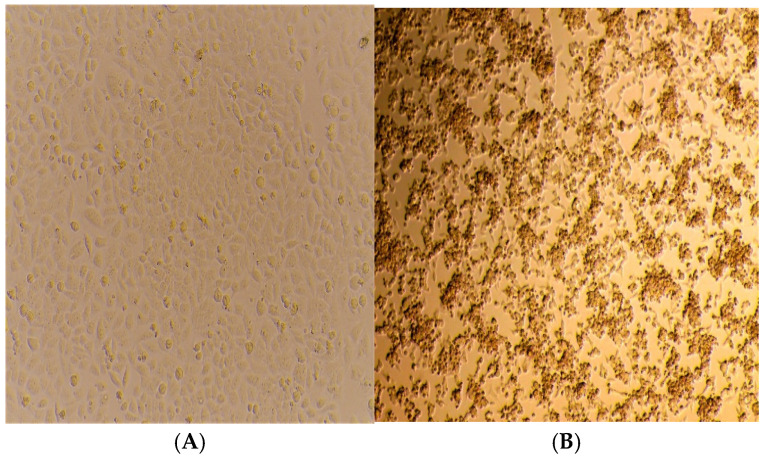
Isolation of CV-A24v in Hela cell line: (**A**) appearance of normal HeLa Cell line; (**B**) HeLa cell line showing 80% CPEs on day 5 post-inoculation.

Among the 73 typed CV-A24v specimens, 32 were selected for an attempt at virus isolation. The assay was conducted using the HeLa cell line, cultured within a 24-hour timeframe. Blind passages in duplicates were carried out from P-1 to P-5. Figure 3A,B depict samples that consistently showed morphological changes in the form of CPEs, noted from day 4 onwards. The most pronounced CPEs comprised cell rounding, cell aggregation into clumps, increased granularity due to the formation of inclusion bodies, and cell detachment from the substrate. Cells exhibiting more than 90 to 95% CPEs were harvested. Confirmations of the viral isolates were performed by subjecting P-5 viral lysate to RT-qPCR. Six CV-A24v isolates were thus obtained.

### 3.3. Confirmation of Isolates by q-PCR

Extraction of RNA from the viral lysate of P-5 was performed using a viral RNA isolation kit (QIAamp viral RNA mini kit, Hilden, Germany). The RNA was then processed using RT-qPCR. The six isolates of 2023 along with the two isolates of 2009 (previously isolated, not reported) were subjected to RT-qPCR to confirm the positivity for EV. The Ct value data for CV-A24v isolates were in the range of 14 to 26 as provided in the tabular form (Table 1).

### 3.4. Whole-Genome Sequencing by NGS

In order to understand the recent molecular trends of CV-A24v viral strains, the eight CV-A24v isolates were processed for whole-genome sequencing using NGS. The genome recovery of the eight isolates after sequencing ranged from 96.88% to 99.95%. The percentage of genome retrieved of CV-A24v along with the total and relevant reads of each of the isolates is listed in Table 2. The full genome sequences of the eight isolated viral strains obtained by NGS were submitted to the GenBank Database, and the accession numbers generated are from PQ095934 to PQ095941 (Table 2).

### 3.5. Phylogenetic Analysis

**Figure 4:**
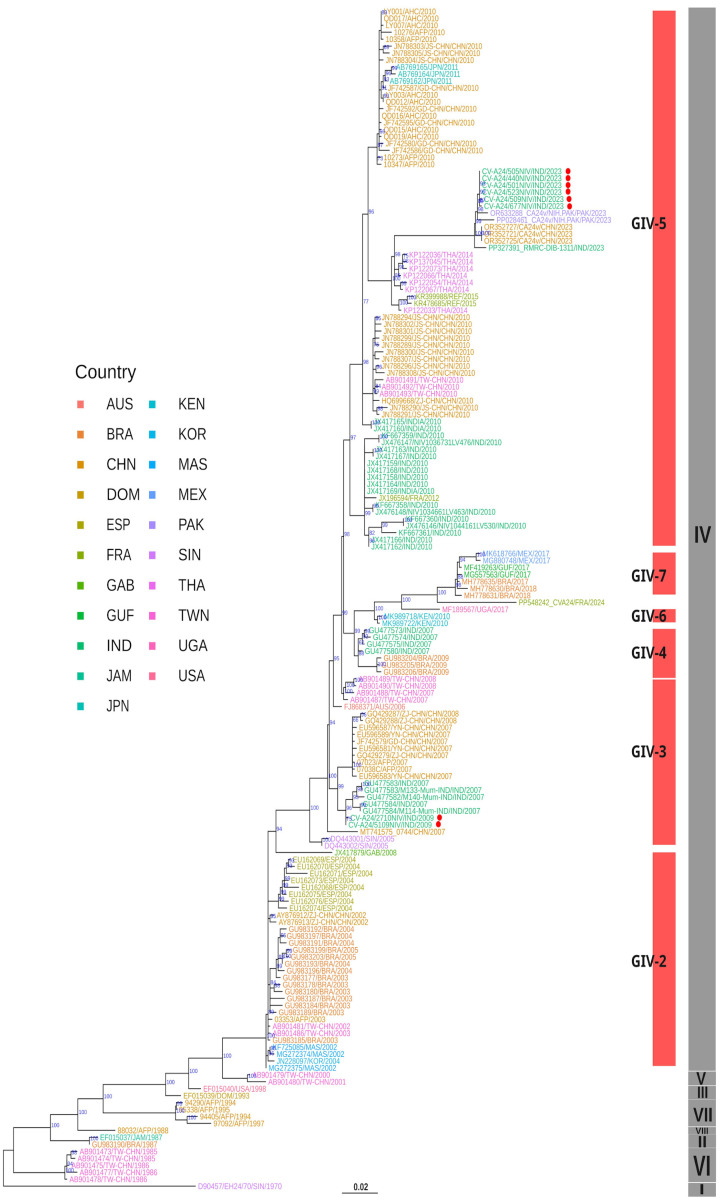
Maximum likelihood phylogenetic analysis based on 171 VP1 sequences of CV-A24v. The tree tips are color-coded according to the countries of origin for the CV-A24v viral isolates. Indian isolates from 2009 and 2023 are highlighted with red circles. Information regarding the CV-A24v genotype is displayed in a grey vertical bar, while the sub-genotype is indicated with a red vertical bar positioned in front of the phylogenetic tree.

To understand the classification, origin, and evolution of the six CV-A24v strains isolated in 2023 from the conjunctivitis outbreak across India, all the sequences of the CV-A24v isolates were compared with the homologous sequences available from the rest of the world based on the whole-genome, VP1, and VP2 datasets. Initially, a maximum likelihood (ML) phylogenetic tree was constructed based on 171 VP1 sequences, which delineated multiple subclades consistent with previously recognized genotypes I–VIII (Figure 4).

Notably, the six Indian isolates of 2023 were distributed across two distinct subclades within GIV. The two sequences from the 2009 outbreak clustered within the GIV-3 subclade, alongside previously identified strains from India dating back to 2007. In contrast, the six CV-A24v isolates from 2023 formed a monophyletic cluster within GIV-5, demonstrating a close evolutionary relationship with viral strains from China and Pakistan, suggesting a shared common ancestor. Further analysis also revealed previously unassigned sub-clusters within GIV, annotated using the TreeCluster tool [21]. This effort led to the identification of two novel subclades: GIV-6 and GIV-7 (Supplementary Table S1) within genotype IV, enhancing our understanding of the evolutionary trajectory of CV-A24v.

**Figure 5:**
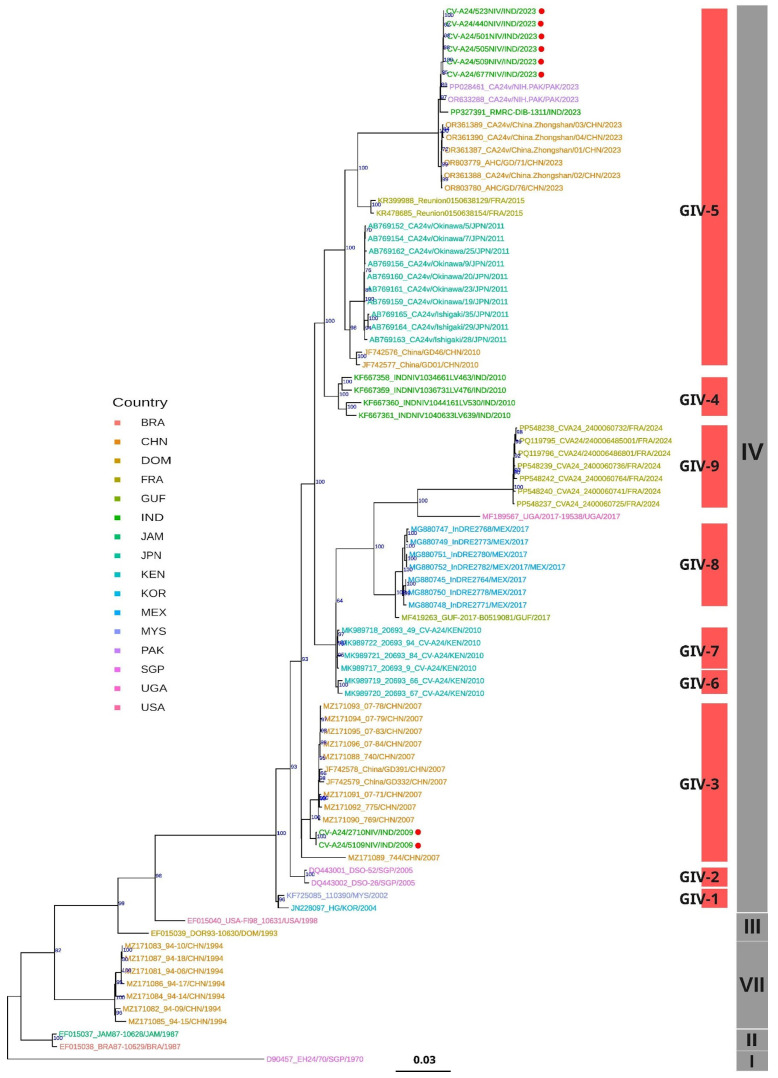
Maximum likelihood phylogenetic analysis based on 84 whole-genome CV-A24v sequences. The tree tips are color-coded according to the countries of origin for the CV-A24v viral isolates. Indian isolates from 2009 and 2023 are highlighted with red circles. Information regarding the CV-A24v genotype is displayed in a grey vertical bar, while the sub-genotype is indicated with a red vertical bar positioned in front of the phylogenetic tree.

ML-based phylogenetic analysis of 84 whole-genome sequences (Figure 5) and 92 VP2 sequences (Figure 5) revealed that the Indian viral strains from both 2009 and 2023 grouped within genotype IV. The 2009 isolates clustered in GIV-3 and 2023 isolates in GIV-5 subclades, indicating tree topologies concordant with the VP1 phylogeny (Figure 3). BLAST similarity analysis of whole-genome sequences showed that the 2009 Indian isolates shared 99.02% similarity with a 2009 strain from China (MZ171090), while exhibiting only 86.31–86.32% similarity with the Singapore ancestral prototype strain D90457 [23] (Supplementary Table S2).

Conversely, the 2023 isolates displayed 99.20 to 99.48% similarity with the sequence of the Chinese isolate, OR361388, and 99.42 to 99.50% similarity with the sequence of Pakistan strain OR633288.

Mutation analysis with the whole-genome sequences was performed against the Singapore 1970 strain D90457 as a prototype reference strain [23]. The analysis revealed a total of nine non-synonymous changes, spanning across the genome, in a few of the Indian strains of this study (Table 3). This highlights the ongoing evolution and adaptation of the virus in the country. Interestingly, it was observed that the 2009 sequences exhibited unique mutations in the VP1 and 3D regions, demonstrating the distinct characteristics of the specific time period. On the other hand, the sequences of 2023 displayed unique mutations in the VP3, 2C, and 3D regions that have not been reported in previous analyses, indicating newer mutations beyond 2009.

**Figure 6:**
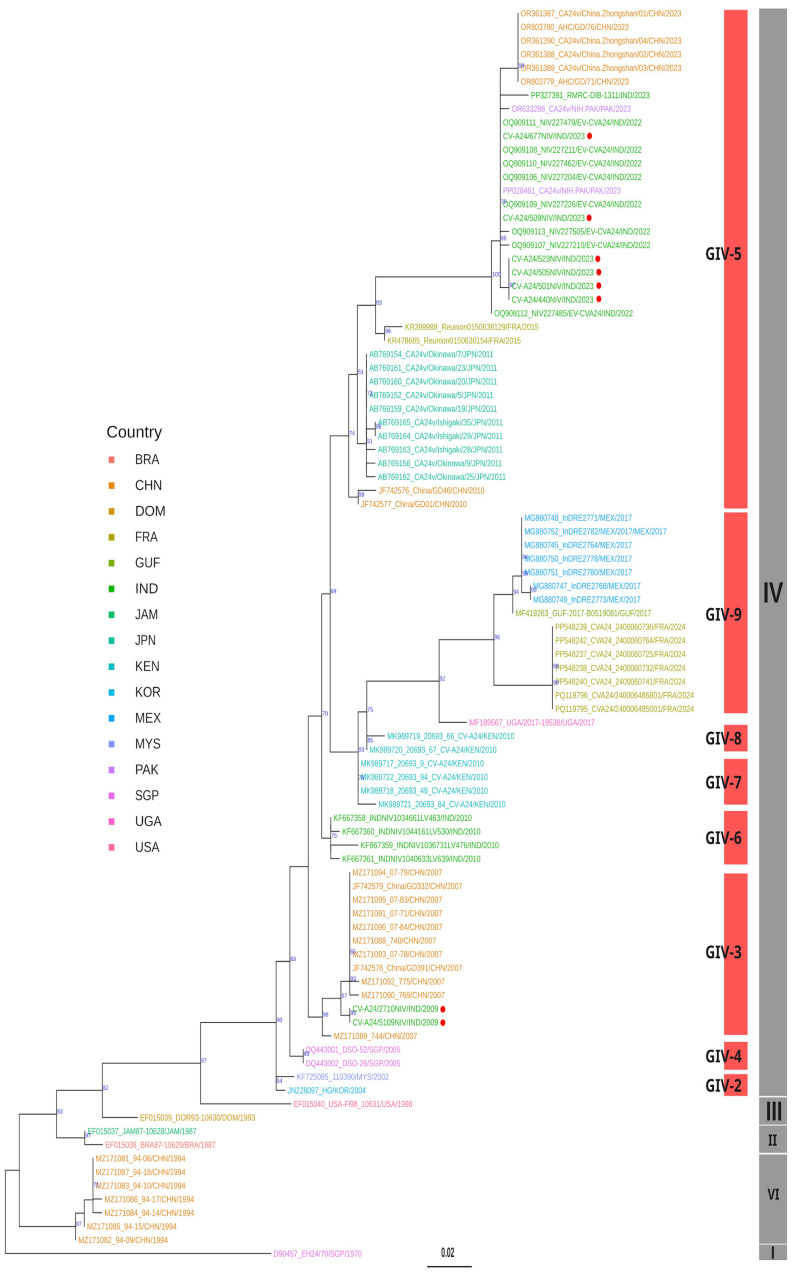
Maximum likelihood phylogenetic analysis based on 92 VP2 sequences of CV-A24v. The tree tips are color-coded according to the countries of origin for the CV-A24v isolates. Indian isolates from 2009 and 2023 are highlighted with red circles. Information regarding the CV-A24v genotype is displayed in a grey vertical bar, while the sub-genotype is indicated with a red vertical bar positioned in front of the phylogenetic tree.

Phylogenetic analysis of partial VP2 sequences (Figure 6) revealed the clustering of the 2023 Indian CV-A24v isolates alongside the sequences of the 2022 viral strains from China and Pakistan within the GIV-5 subclade. Notably, the Indian viral strains from 2022 formed an outgroup within this subclade, suggesting a potential transmission route from India to China and Pakistan during the 2023 outbreak. Additionally, TreeCluster-based analyses of whole-genome and VP2 gene datasets revealed four additional subclades within GIV (GIV-C6 to GIV-C9), highlighting greater phylogenetic diversity than that previously reported (Supplementary Table S1). In conclusion, the phylogenetic analysis confirmed that the Indian CV-A24v viral strains isolated in 2009 and 2023 belong to the genotype IV clade, thus providing new insights into their evolutionary history and geographic spread.

### 3.6. Molecular Clock and Phylogeography Analysis

To estimate the evolutionary timescale of CV-A24v strains, evolutionary analysis was performed using VP1 nucleotide sequences with a Bayesian Markov Chain Monte Carlo (MCMC) approach in BEAST. The analysis suggested that CV-A24v was in circulation with the most recent common ancestor (tMRCA) having evolved around 1968 in Singapore, with a posterior probability of 0.87 (95% HPD). The mean molecular evolutionary rate was calculated at 5.463 × 10⁻⁴ substitutions/site/year (95% HPD) (Supplementary Table S2). The maximum clade credibility (MCC) phylogenetic tree (Figure 6) revealed distinct clustering into genotypes GI to GVIII, consistent with the ML tree (Figure 4).

**Figure 7:**
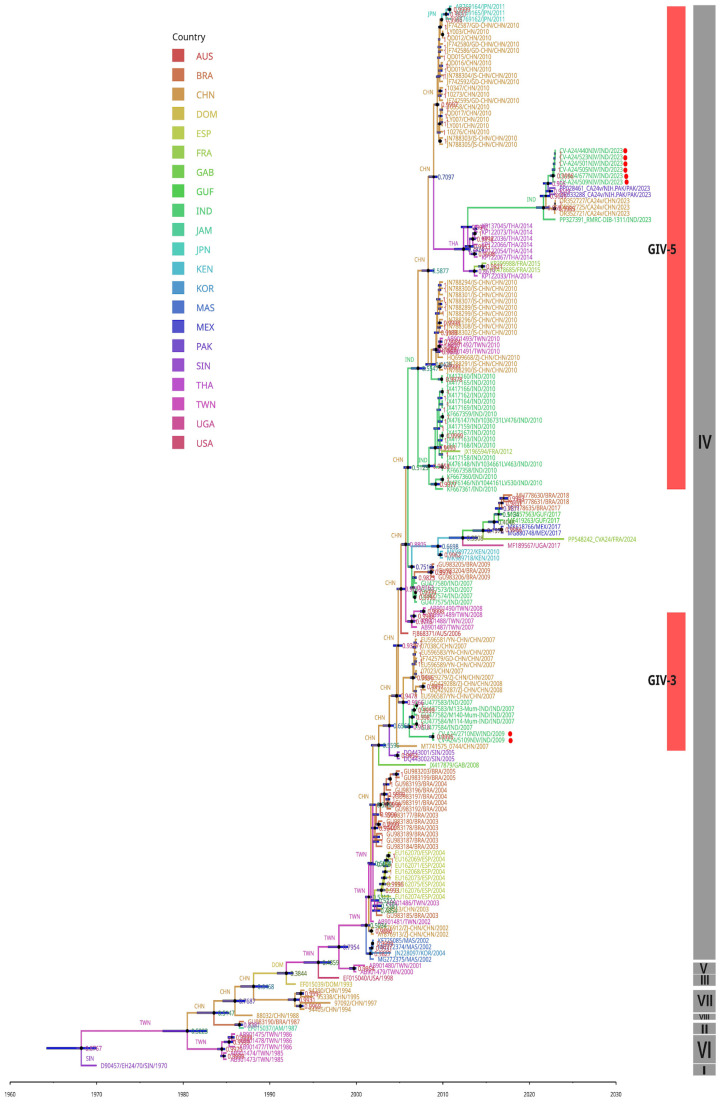
MCC phylogenetic analysis based on 171 VP1 sequences of CV-A24v. The branches and tips of the tree are color-coded according to the countries of origin for the CV-A24v isolates. Indian viral isolates from 2009 and 2023 are highlighted with red circles. Information regarding the CV-A24v genotype is displayed in a grey vertical bar, while the sub-genotype is indicated with a red vertical bar positioned in front of the phylogenetic tree. The node labels are according to country posterior probability and ancestral states. TMRCAs values at 95% HPDs are indicated with bars at nodes of the tree.

The investigation of ancestral states, particularly for the GIV genotype, indicated a transmission event from Taiwan (posterior probability: 0.50) to other regions. The 2009 Indian viral isolates belonging to GIV-3 are estimated to have evolved from strains in China (posterior probability: 0.90). Conversely, the 2023 Indian viral isolates, classified as GIV-5, likely originated from Thailand (2014) (posterior probability: 0.90), followed by subsequent transmissions from India to China and Pakistan, as India was further noted to be the ancestral source for the GIV-5 sub-subclade (posterior probability: 0.99) (Figure 7).

**Figure 8:**
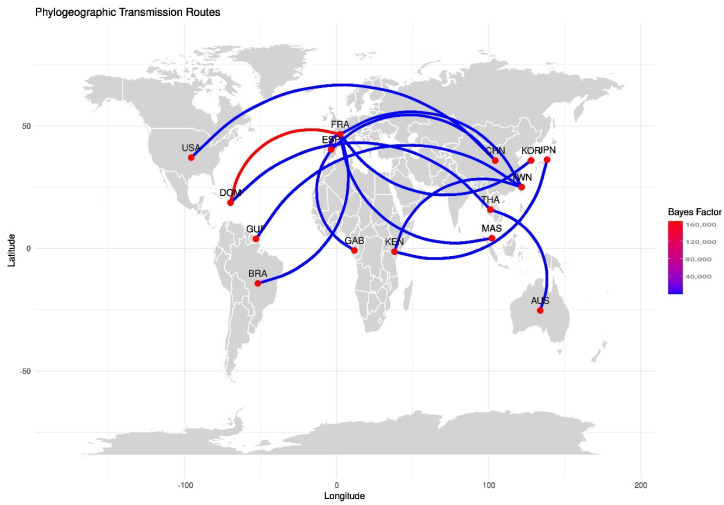
Geographic distribution and transition rates of CV-A24v isolates between countries, with supported posterior probabilities greater than 0.5. The figure represents the transmission routes using SPREAD software v0.9.7.1 using the world map [22]. Lines between countries indicate the possible transmission routes of CV-A24v. Colored lines indicate BF values and the posterior probability.

A discrete phylogeographic reconstruction of CV-A24v using SPREAD revealed significant global transmission routes [22]. Outputs with Bayes factor values greater than 10 and probabilities exceeding 0.5 were considered for visualizing the geographic spread (Figure 8) (Supplementary Table S2). The key findings include strong transmission potential of the virus from France to regions such as the Dominican Republic, Spain, Malaysia, and the Republic of Korea. Intercontinental spread of CV-A24v was also observed, with viral transmission links between China and the USA, and also Taiwan and Kenya. Moderate transmission likelihood was noted between Brazil and France, as well as Kenya and Japan. Notably, no significant transmission links involving India were detected in the migration pathway analysis.

## 4. Discussion

As per previous reports of AHC outbreaks from Acera, Ghana, and Singapore during 1969-1970, two *picornaviruses*, EV-D70 and CV-A24v, were isolated and identified as the etiology of the outbreaks [5,24,25]. In 1971, during the AHC outbreak in Hong Kong, both EV-D70 and CV-A24v viral strains were isolated, indicating that these viruses had a broader geographical distribution. In 1975, Singapore and Sri Lanka highlighted the potential global spread of CV-A24v [26]. The AHC outbreak in 1986 in American Samoa, with CV-A24v as the etiological agent, was the first outbreak outside Southeast Asia and in the Indian subcontinent [27]. Several AHC outbreaks occurred in Okinawa Prefecture, Japan, in 1985 and 1986, representing the extensive spread of the virus to new geographical areas [28]. Brazil reported several AHC outbreaks in 1987 associated with CV-A24v, originating from Asia, and such transmissions continued to influence Brazilian outbreaks in 2003 and 2009 [29]. Further, the emergence of a novel GIV strain in Taiwan during the 2000–2002 and 2006–2007 outbreaks was suggested to have its origin in Marseille, France [30]. 

Environmental and host factors play a crucial role in the evolution and spread of CV-A24v. Environmental conditions, such as temperature, humidity, and sanitation, can influence virus transmission and survival, particularly in densely populated areas with limited hygiene practices. In India, both environmental and host factors significantly influence the evolution and spread of CV-A24v. The country’s tropical climate creates favorable conditions for the survival of the virus and its transmission, particularly in crowded urban areas with poor sanitation. Host factors, such as varying levels of immunity in the population, play a key role in the viral spread, as younger individuals or those with weakened immune systems are more susceptible. Additionally, the frequent movement of people due to travel and festivals facilitates the rapid transmission of the virus, leading to recurrent outbreaks across the country.

Since 1970, India has experienced several sporadic outbreaks of conjunctivitis each year across the country, but many of these outbreaks or cases often go unnoticed due to a weak surveillance system, the widespread use of over-the-counter drugs, and the self-limiting nature of the disease. Compared to the data from the last three years, undocumented cases may be attributed to the SARS-CoV-2 pandemic. The surge in conjunctivitis cases over a short period could be linked to immunosuppression resulting from COVID-19 or a loss of herd immunity to CV-A24v. 

The widespread 2023 outbreak of AHC in this study was noted to be of a Pan-India nature. Genomic sequencing of VP1 and 3C region from ocular swabs confirmed CV-A24v as the etiological agent belonging to a new lineage cluster related to GIV-C5 [31]. Genomic sequencing of ocular swabs from the Mexico outbreak revealed that the isolates belong to a new clade related to genotype IV of CV-A24v [32]. Recently, in 2023, Pakistan experienced a surge in AHC cases, and the whole-genome sequencing of CV-A24v revealed genotype GIV with notable mutations in the 3D and VP1 regions. Further, phylogenetic analysis indicated higher homology with isolates from Zhongshan, China, 2023 [9]. 

The dynamic genetic landscape of CV-A24v highlights the need for continuous surveillance and effective research for the management of AHC outbreaks. To understand the existing circulating pattern of viral strains causing the widespread AHC outbreak of 2023, this study aimed to isolate the identified CV-A24v viral strains and explore their origin and evolution by using NGS-based whole-genome sequencing for the 2023 outbreak strain and the strain from an earlier outbreak in 2009. Phylogenetic analyses, based on whole-genome sequencing, as well as VP1 and VP2 genes, revealed a clear classification into eight genotypes GI to GVIII, affirming previous findings [9,15,31,33]. Genotype IV emerges as the largest and most diverse group, further subdividing into five subclades [31,33,34]. However, the implementation of the TreeCluster tool in this study revealed additional novel subclades within GIV, namely GIV-6 and GIV-7. The CV-A24v viral strains responsible for the 2009 and 2023 outbreaks were found to be genetically distinct within the GIV genotype [Figure 3, Figure 4 and Figure 5]. The ML and MCC VP1 trees revealed the 2009 Indian viral isolates as belonging to the GIV-3 subclade, showing a close relationship to the 2007 isolates from India and indicating a potential local transmission event. In contrast, the 2023 viral isolates clustered within the GIV-5 subclade, exhibiting close genetic relatedness to recent viral strains from China and Pakistan with potential migration from Thailand (2014) and France (2015) [31,33]. These findings suggest probable cross-transmission of viral strains occurring due to globalization, international travel, and trade. The findings also align with earlier data from India where CV-A24v was responsible for causing different outbreaks in 1986, 1988, 1994, 1999, 2003, 2009, and 2023, as determined through virus isolation and sequencing [8,10,35,36,37,38,39]. 

The mutation analysis revealed unique evolutionary signatures in the Indian CV-A24v viral strains. Nine mutations were identified across the genomes of individual viral strains of 2009 and 2023 when compared to the Singapore prototype strain D90457 (Table 3) [23]. The occurrence of the mutations highlights the ongoing evolution of the virus, with unique mutations in each outbreak pointing to specific adaptive changes over time. The functional significance of these mutations in terms of pathogenicity, however, needs to be studied. On the other hand, conserved regions within different genotypes may give insights into the design of potential candidate targets for vaccine development. So, as no specific antiviral drugs have been approved against viral agents causing AHC, the findings from the mutational analysis may also be useful in drug design efforts.

Furthermore, the molecular clock analysis using VP1 gene sequences estimated a timeline for CV-A24v origin around 1968, with a mutation rate of 5.463 × 10⁻^3^ substitutions/site/year, congruent with previous studies [15]. This substitution rate, compared to related viruses such as Echovirus 6 (7.0 × 10^−3^ substitutions/site/year), Echovirus 14 (9.2 × 10^−3^ substitutions/site/year), Coxsackievirus B1 (7.0 × 10^−3^ substitutions/site/year), and EV-A71 (7.2 × 10^−3^ substitutions/site/year) [40,41,42,43], suggests that specific growth environments may restrict the mutational rates of these viruses within the *Picornaviridae* family [Figure 7].

The phylogeographic analysis of Indian isolates with respect to strains from other countries demonstrated distinct transmission pathways of the 2009 and 2023 viral strains, providing new insights into the evolutionary dynamics of CV-A24v and its spread across different geographical regions. The findings highlight the importance of region-specific preventive measures and surveillance, which may also have implications for global health policies and travel regulations. Collectively, these findings can help in vaccine strategies, enhance outbreak preparedness, and aid in the development of diagnostic tools to control future outbreaks.

## 5. Conclusions

The etiology of the AHC outbreak that occurred in 2023 was identified as CV-A24v and the virus was successfully isolated. To understand the origin of the outbreak, the genetic composition of the viral agent, and the evolutionary pattern, phylogenetic studies based on whole-genome sequencing, VP1, VP2, and 3C were undertaken. The isolated CV-A24v viral strains from the 2009 and 2023 AHC outbreaks were both assigned to the GIV clade, but genetically distinct. Local transmission events may have been responsible for the 2009 AHC outbreak, which was shown to belong to the GIV-3 subclade, with a close relationship to the 2007 isolates from India. In contrast, the viral isolates from 2023, clustering within the GIV-5 subclade and exhibiting close genetic relatedness with the recent viral strains from China and Pakistan, with potential migration from Thailand and France, emphasize the role of global transmission routes in the spread of the virus.

## Figures and Tables

**Table 1:** Comparative Ct values of clinical specimens and their isolates in ascending order.

Well No.	Fluorescent Dye	Target	Sample ID	Ct Values of Clinical Specimens	CT Values of Isolates
E06	FAM	PAN EV	NC	-	-
* C06	FAM	PAN EV	CV-A24v/5106	-	14.52
* D06	FAM	PAN EV	CV-A24v/2710	--	17.17
B04	FAM	PAN EV	CV-A24v/677	27.62	17.63
D03	FAM	PAN EV	CV-A24v/505	28.40	21.93
E01	FAM	PAN EV	CV-A24v/446	26.01	24.59
B01	FAM	PAN EV	CV-A24v/501	25.24	24.81
D01	FAM	PAN EV	CV-A24v/523	26.76	25.18
G02	FAM	PAN EV	CV-A24v/509	26.30	26.07
F06	FAM	PAN EV	PC		31.08

* Conventional PCR was performed for these clinical specimens collected in 2009.

**Table 2:** NGS data of CV-A24v isolates including sample ID, total reads, relevant reads, and genomic length (mapped with Singapore reference strain D90457).

SL. No.	Sample ID	Total Reads	Avg. Coverage	Relevant Reads	Consensus Length	Genome Retrieved Percentage	Accession Number
1	CVA-24v/5109	3,087,154	35,242.36	2,067,551	7456	99.93	PQ095934
2	CVA-24v/2710	5,586,990	59,825.40	3,870,238	7457	99.95	PQ095935
3	CV A-24v/677	161,094	536	30,156	7211	96.65	PQ095936
4	CV A-24v/505	2,055,898	3579.94	193,627	7455	99.92	PQ095937
5	CV A-24v/440	1,828,954	374.72	20,146	7303	97.88	PQ095938
6	CV A-24v/501	1,760,594	363.29	19,637	7228	96.88	PQ095939
7	CV A-24v/523	2,858,114	539.44	29,495	7327	98.20	PQ095940
8	CV A-24v/509	2,902,554	377.45	20,570	7233	96.94	PQ095941

**Table 3:** Identification of unique mutations in CV-A24v 2009 and 2023 viral strains isolated in India against the reference, 1970 Singapore strain D90457.

S. No.	Gene/ Region	CV-A24 Isolates		Nucleotide Substitution	Amino Acid Substitution Position	Amino Acid Substitution	Substitution Type
1.	VP3	CV-A24/501NIV/IND/2023	1934	T→C	395	M→T	Non-Synonymous
2.	VP3	CV-A24/505NIV/IND/2023	1940	tca→ttg	397	S→L	Non-Synonymous
3.	VP3	CV-A24/677NIV/IND/2023	2432	T→C	561	V→A	Non-Synonymous
4.	VP1	CV-A24/2710NIV/IND/2009	2800	aag→gaa	684	K→E	Non-Synonymous
5.	2C	CV-A24/501NIV/IND/2023	4872	C→G	1374	F→L	Non-Synonymous
6.	2C	CV-A24/501NIV/IND/2023	4873	gcc→cct	1375	A→P	Non-Synonymous
7.	3D	CV-A24/505NIV/IND/2023	6056	gtt→gcg	1769	V→A	Non-Synonymous
8.	3D	CV-A24/2710NIV/IND/2009	6333	G→T	1861	E→D	Non-Synonymous
9.	3D	CV-A24/5109NIV/IND/2009	6333	G→T	1861	E→D	Non-Synonymous

## Data Availability

The raw data supporting the conclusions of this manuscript will be made available by the authors, without undue reservation.

## Supplementary Materials

The following supporting information can be downloaded at https://www.mdpi.com/article/10.3390/v17030371/s1: Legends of supplementary tables. Table S1: This table lists the identified clusters for VP1, VP2, and whole-genome sequences using TreeCluster; Table S2: This table represents the all-vs.-all BLAST results using complete genome sequences; Table S3: This table illustrates the transmission routes with posterior probability and Bayes Factor values using SPREAD software.
